# Periodontal and Peri-Implant Diagnosis: Current Evidence and Future Directions

**DOI:** 10.3390/diagnostics14030256

**Published:** 2024-01-25

**Authors:** Francesco D’Ambrosio

**Affiliations:** Department of Medicine, Surgery and Dentistry “Schola Medica Salernitana”, University of Salerno, Via S. Allende, 84081 Baronissi, Italy; fradambrosio@unisa.it

Dentistry and periodontology in particular are constantly evolving in terms of both diagnostic and therapeutic tools [1].

Periodontitis is an inflammatory pathology caused by bacteria, which causes the destruction of the periodontium, i.e., the anatomical structure that supports the teeth. To overcome bone loss and, ultimately, tooth loss, however, further diagnostic and therapeutic advancements have been proposed in recent years [2,3,4].

Periodontitis is one of the main causes of tooth loss in industrialized countries and, similarly, peri-implantitis is an irreversible inflammatory disease, which affects dental implants with the consequent loss of osseointegration [4,5].

Furthermore, diagnosing periodontal and peri-implant disease early and eliminating the infection with adequate therapeutic protocols makes it possible to prevent periodontal and pre-implant bacteria present in the oral cavity from entering the bloodstream and influencing systemic diseases such as muscle diseases, intestinal inflammation, metabolic disorders, some types of cancer, Alzheimer’s disease, systemic endocrine diseases such as diabetes, and immune system diseases such as rheumatoid arthritis and atherosclerosis [6,7,8,9].

The most recent diagnostic protocol is based on the recent 2017 classification of periodontal and peri-implant disease [10,11].

According to the 2017 classification, periodontitis is divided into four stages based on severity, and the classification also considers the loss of clinical attachment, the depth of the pockets, the intrabony defects, the involvement of the furcation, the hypermobility of the teeth, and the chewing and oral dysfunction extension (localized or generalized) [12,13,14,15].

Furthermore, the progression rate of periodontal disease has been defined, which can be divided into three categories: slow, moderate, and rapid progression (grades A–C) [14,15].

Risk factors, such as cigarette or vape smoking and diabetes, have been described and are considered as grade modifiers [16,17,18,19].

A periodontal examination is performed through anamnesis, physical examination, radiographic examination, and laboratory tests [20].

Medical history, highlighted by the 2017 classification, is used with the aim of searching for a series of factors that influence the onset and progression of periodontitis, including environmental exposures such as smoking and the use of certain drugs; the presence of systemic diseases such as diabetes; genetic predispositions; and hormonal changes [17,18,21].

An objective examination is essentially based on the observation of periodontal tissues and on periodontal probing, which is an essential clinical maneuver for the diagnosis of periodontal disease, as it allows for a differential diagnosis between gingivitis and periodontitis to be made, as well as for the estimation of the loss of clinical attachment [11,12,22,23,24,25].

A survey is carried out with a standardized periodontal probe instrument which is used to measure depth in millimeters; it must be placed between the tooth and gum at the correct angle and pushed with a force of approx. 25–30 g until the bottom of the sulcus or pocket is reached [26,27].

This probe allows one to measure the depth of periodontal pockets and recessions, to identify the involvement of furcations, to detect the presence of bleeding on probing, and to detect the presence of tartar and the subgingival location of overflowing restorations.

The survey must be carried out by sliding the probe along the entire length circumference of each tooth, but while this will be recorded, the standard position is normally six sites per tooth [26,27,28].

There are also "simplified" methods of recording the clinical findings obtained with the survey. One of these modalities is the PSR, Periodontal Screening and Recording [28].

Intraoral radiological examinations, performed in cases or in areas where they represent a useful complement to the objective examination, allow for the the acquisition of new information for use in diagnoses and/or treatment plans [28,29].

Laboratory tests can be a complement to a diagnosis in cases of severe periodontitis or in cases of periodontitis associated with systemic pathologies [28,29].

Microbiological tests to determine the bacteria responsible for periodontal diseases; hematological tests for the study of the number and functionality of polymorphonuclear cells and lymphocytes; and tests to identify genetic susceptibility to periodontitis are among the main laboratory tests available [26,27,28,29].

For the treatment of periodontitis (stages I, II, and III), a gradual therapeutic approach has been described which should vary depending on the stage of the disease [28].

Primary management is based on non-surgical treatment through the removal of periodontal bacteria through oral hygiene, scaling, and root planning (SRP) [26,27,28,29].

This first phase aims to create a periodontal environment that allows the periodontal tissues to heal and enables the possibility of the long-term preservation of the teeth affected by the disease [28,29,30].

Non-surgical periodontal therapy has a long history and is performed using both manual and mechanical instruments to decontaminate root surfaces by removing bacterial plaque, tartar, and contaminated root cement [29].

However, numerous other approaches have been described in the literature to increase the effectiveness of non-surgical therapy, such as ozone, the addition of erythritol-chlorhexidine powder, probiotics, laser therapy, hyaluronic acid, omega-3, omega-6, and drugs such as antibiotics or other medications [30,31,32,33,34,35,36,37,38,39,40,41,42,43,44,45].

The importance of maintaining adequate oral hygiene and the adequate control of periodontal disease has been proven to be of paramount importance during the COVID-19 pandemic, as severe forms of periodontal disease have been linked to more severe forms of COVID-19 [45,46,47,48,49,50,51].

Considering this, progress in diagnosis and therapy in periodontal and peri-implant disease with the implementation of new therapeutic aids will constitute the basis for the future therapeutic approach to periodontal and peri-implant disease.
